# Exogenous Nucleotides Antagonize the Developmental Toxicity of Ethanol *In Vitro*

**DOI:** 10.1155/2013/204187

**Published:** 2013-11-11

**Authors:** Jie Zhao, Jia-Xi Zhao, Ya-Jun Xu

**Affiliations:** ^1^Department of Nutrition and Food Hygiene, School of Public Health, Peking University, No. 38 Xueyuan Road, Haidian District, Beijing 100191, China; ^2^Beijing Key Laboratory of Toxicological Research and Risk Assessment for Food Safety, Peking University, Beijing 100191, China

## Abstract

The objective of this study was to assess whether nucleotides supplementation *in vitro* could suppress ethanol-induced developmental toxicity in mouse. The models of whole embryo culture (WEC) and midbrain (MB) cell micromass culture were used in this study. In WEC system, exposure to 4.0 mg/mL ethanol for 48 h yielded various developmental malformations of the mice embryos. Nucleotides supplementation (0.16, 0.80, 4.00, 20.00, and 100.00 mg/L) improved the growth parameters to some extent, and the protective effects peaked at 4.00 mg/L. In MB cell micromass culture system, exposure to 4.0 mg/mL ethanol for 5 days resulted in suppression of proliferation and differentiation. Supplementation of nucleotides (0.16, 0.80, 4.00, 20.00, and 100.00 mg/L) showed some protective effects, which peaked at 4.00 mg/L, too. The present research indicated that nucleotides supplementation might be of some benefit in the prevention of ethanol-induced birth defects; however, appropriate dosage requires attention.

## 1. Introduction

 A wide array of fetal disorders can be induced by alcohol consumption during pregnancy, from subtle changes in intelligence to severe mental retardation and physical disability [1]. The most severe is known as fetal alcohol syndrome (FAS), the major clinical features of which are pre- and postnatal growth retardation, central nervous system (CNS) dysfunction, and craniofacial malformations, together with some other minor abnormalities. Most of the damages cannot be reversed totally and bring about heavy burdens to the suffered families. Besides, there is no so-called “safe dose” of alcohol ingestion in pregnancy. Even low levels of prenatal ethanol exposure may exert a significant impact upon later development of fetus [2]. Although the knowledge of alcoholic beverage drinking during gestation may jeopardize the offspring is well known, the prevalence of FAS is still increasing worldwidel. Especially in some developing countries where the social status of women is improving and females are attending more and more social activities than before, the odds of unintentional drinking during early pregnancy (the first trimester when many women may be not aware of their pregnancy) is increasing rapidly [3].

Nucleotides are structural units of nucleic acids, RNA and DNA, and are essential compounds in the energy transfer systems. Therefore, it has been assumed that they play an important role in carbohydrate, lipid, protein, and nucleic acid metabolism and as modulators of many neonatal physiological functions [4]. Nucleotides requirement of pregnant women is much more than that of normal people. However ethanol ingestion decreases nucleotides retention for tissues through an increase in bases degradation, which results in nucleotides deficiency in pregnant women and insufficient nucleotides supplies for fetus. On the other hand, ethanol can traverse placenta barrier and directly disturb the nucleotides metabolism of the developing fetus, leading to damages to fetal growth and development. The endogenous supply of nucleotides is maintained through *de novo *synthesis and salvage pathway. Our previous research has pointed out that supplementation of provider of one-carbon unit, necessary material of the *de novo *synthesis of nucleotides, can alleviate ethanol-induced developmental toxicity in mice fetuses [5]. However compared with salvage pathway, *de novo *synthesis of nucleotides is energy demanding and cannot be achieved in some important organs, such as central nervous system, skeleton, heart, and lung. Salvage pathway is relatively energy saving. Considering the limitation of *de novo* synthesis of nucleotides under stress conditions such as impaired immunity, liver injury, and rapid growth period [6], we hypothesized that enhancement of salvage pathway by supplementing exogenous nucleotides might be an effective and economic way to antagonize prenatal ethanol-induced development toxicity. The present investigation utilized a whole embryo culture system and a micromass test that are recommended by European Centre for the Validation of Alternative Methods (ECVAM) as alternative methods for developmental toxicity study.

## 2. Materials and Methods

### 2.1. Chemicals

5′-nucleotides (NT) powder of analytical grade (>99% pure), derived from brew yeast RNA, was provided by Zhen-Ao Biotechnology (Dalian, China). The proportion of 5′AMP : 5′CMP : 5′GMPNa_2_ : 5′UMPNa_2_ in the powder was 22.8% : 26.6% : 30.2% : 20.4%. Anhydrous ethanol of analytical grade was purchased from Beijing Chemical Company (Beijing, China).

### 2.2. Animals

C57BL/6J mice, aged 12 weeks, were supplied by Vital River Laboratory Animal Technology Co. Ltd (Beijing, China). All mice were maintained in a temperature- and humidity-controlled animal facility with a 12/12 h light/dark cycle and provided with food and water ad libitum throughout the study. Dams were caged with sires overnight and copulation was assessed the following morning by the presence of a vaginal plug to signify gestation day (GD) 0. The use of animals in this research was in accordance with the guidelines for animal research of Peking University.

### 2.3. Whole Embryo Culture Test

#### 2.3.1. Embryo Isolation and Culture

*In vitro* postimplantation whole embryo culture was carried out according to the method developed by New [7] and adapted by Van Maele-Fabry et al. [8]. On GD 8.5, pregnant mice were killed by cervical dislocation and the uteri were removed into sterile Hank's solution. Embryos displaying 3–5 pairs of somites were selected for culture. After removal of the deciduas and Reichert's membrane, those embryos with intact yolk sacs and ectoplacental cones were placed randomly into sterilized culture bottles (3 embryos/bottle), each containing 3 mL culture medium. Culture medium was 100% male rat serum that was immediately centrifuged, heat-inactivated (56°C for 30 min), filter-sterilized, and supplemented with 100 unit/mL penicillin G and 100 *μ*g/mL streptomycin. The embryos were cultured for 48 h at 37.5 ± 0.5°C, rotated at 40 rev/min. The culture bottles were gassed initially for 2.5 min with 5% O_2_ : 5% CO_2_ : 90% N_2_. Subsequent regassing for 2.5 min occurred at 20 h (20% O_2_ : 5% CO_2 _ : 75% N_2_) and 30 h (40% O_2_ : 5% CO_2_ : 55% N_2_). At the end of the 48 h culture, all embryos were removed from the culture bottles and put into prewarmed Hank's solution for evaluation. 

The experimental groups included: (i) normal control group, (ii) ethanol control group, (iii) five nucleotide intervention groups. Ethanol was added at 4.0 mg/mL as the final concentration in all groups except the normal control. The five nucleotide intervention groups were additionally exposed to 0.16, 0.80, 4.0, 20.0, and 100.0 mg/L nucleotides, respectively.

#### 2.3.2. Morphological Evaluation

At the end of the 48 h culture period, cultures were terminated, and all embryos were removed from the culture bottles and placed in prewarmed Hank's solution for morphological evaluation. Morphological evaluation of embryos was conducted under a stereomicroscope using the morphologic scoring system by Van Maele-Fabry et al. [8]. Only viable embryos (presence of yolk sac circulation and heartbeat) were examined. The growth and morphological features assessed included embryonic flexion, heart, tail neural tube, cerebral vesicles (fore-, mid-, and hindbrain), visual, auditory, and olfactory systems, branchial arch, limb buds (forelimb and hindlimb), crown-rump length (CRL), and head length (HL). Besides, yolk sac circulation scores and diameter of visceral yolk sac (VYS) of each viable embryo which reflected development of VYS were also assessed in our study. All of the scoring was carried out by a trained observer who was unaware of the treatment.

### 2.4. Midbrain (MB) Cell Culture—Micromass Test

#### 2.4.1. MB Cell Isolation and Culture

On day 12 of gestation, time-pregnant mice were sacrificed by cervical dislocation. Embryos were removed from uterus and transferred to sterile warm (37°C) Hank's balanced salt solution. MB was isolated, washed three times, and incubated in sterile warm (37°C) calcium- and magnesium-free phosphate buffered saline (PBS) for 20 min. PBS was then replaced with 0.5% trypsin in PBS for 10 min at 37°C, and trypsin action was terminated by adding medium (Ham's F12 nutrient mixture : fetal bovine serum : L-glutamine : Pen/Strep : 88 : 10 : 1 : 1). Cells in certain volume of medium were dissociated by repetitive flushing through pipette with 200 ll tip. Single cell suspension was censured by passing the suspension through sterile stainless steel 200 mesh filter. Cells were counted in haemocytometer and adjusted to 5 × 10^6^ cells per mL. 10 *μ*L drop of cell suspension was plated in the center of each well of 96-well microplate for assessment of proliferation, and 20 *μ*L drop of cell suspension was plated in the center of each well of 24-well microplate for assessment of differentiation. Then the microplates with cell drops were placed in incubator for 2-3 h. After that, 200 *μ*L or 2.0 mL medium with or without test chemicals was added into each well of 96-well or 24-well 24 microplate, respectively. 

The experimental groups included: (i) normal control group, (ii) ethanol control group, (iii) five nucleotide intervention groups. Ethanol was added at 4.0 mg/mL as the final concentration in all groups except the normal control. The normal control group received sterile twice-distilled water. The five nucleotide intervention groups were additionally exposed to 0.16, 0.8, 4.0, 20.0, and 100.0 mg/L nucleotides, respectively. The cells were cultured for 5 consecutive days. 

#### 2.4.2. Assessment of Proliferation

Proliferation was measured by 3-(4,5-dimethylthiazol-2-yl)-2,5-diphenyl tetrazolium bromide (MTT) colorimetric assay. The tetrazolium salt was obtained from Sigma, dissolved in PBS at a concentration of 5 mg/mL, and sterilised by filtration. On the end of day 5, a volume of 20 *μ*L MTT solution was added to each well. After a further incubation of 4 h, the medium was aspirated from the wells as completely as possible without disturbing the formazan crystals and cells on the plastic surface. 100 *μ*L DMSO was then added to each well, and the plates were agitated on a plate shaker for 10 min. The optical density was then read at 490 nm on a Bio-Rad Model 550 microplate reader. 

#### 2.4.3. Assessment of Differentiation

MB cell cultures were fixed in 4% formaldehyde and stained using Harries haematoxylin. Images of foci of differentiated cells were captured by CCD Nikon DXM1200F linked to microscope NikonTE2000S. Number (denoted num.) and total area (denoted area) of foci were analyzed by computer image analysis software Image Pro Plus 6.0. 

### 2.5. Statistical Analysis

All statistical analyses were performed with the statistical software package SPSS 13.0 (SPSS Inc., Chicago, IL, USA). Parameters were evaluated with one-way analysis of variance (ANOVA), and data were expressed as mean ± standard deviation (S.D.). All items that exhibited differences then were tested for homogeneity of variances, and the least significant difference (LSD) post hoc test was applied if equal variance existed; otherwise, the Tamhane's T2 test was used. *P* < 0.05 was taken as the level of significance for all analyses.

## 3. Results

### 3.1. Effects of Exogenous Nucleotides on Growth and Morphology Development of Mouse Embryos Exposed to Ethanol *In Vitro*

**Figure 1:**
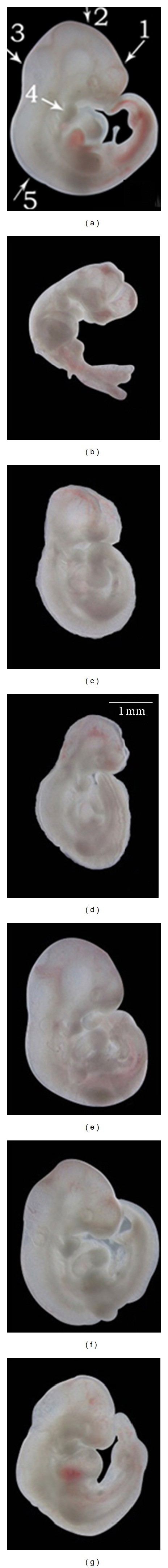
Effect of Exogenous nucleotides on ethanol-induced developmental malformation on mouse embryos cultured *in vitro*. (a) Normal control; (b) ethanol control; (c) 0.16 mg/L; (d) 0.80 mg/L; (e) 4.00 mg/L; (f) 20.00 mg/L; (g) 100.00 mg/L. 1: fore brain; 2: midbrain; 3: hind brain; 4: branchial arch; 5: tail neural tube. Failure of neural folds to fuse in the midline in one or more regions of the forebrain, midbrain, and hindbrain was obvious in ethanol control, 0.16 mg/L nucleotides, and 0.80 mg/L nucleotides intervention groups. 4.00 mg/L nucleotides showed best protective effects. The morphology development became worse at doses above 4.00 mg/L.

CRL and HL were growth parameters and the other parameters listed in Table 1 were morphological development parameters. Statistically significant differences were detected among groups in all the parameters listed. Embryos in normal control group grew well and showed no malformations. The embryos established rapid heartbeat with circulation in the visceral yolk sac and embryos, completed closure of the cranial neural folds, and formed the optic and otic system (Figure 1). 4.00 mg/mL ethanol induced great damages to the development of the embryos, indicated by reduced CRL and HL (*P* < 0.05), as well as defects in central nervous system (CNS), heart, visual and auditory system, branchial arch, and so on (Figure 1). CNS was the main target and the defects included failure of neural folds to fuse in the midline in one or more regions of the forebrain, midbrain, and hindbrain as well as microcephaly, which were consistent with the descriptions of fetal alcohol syndrome.

Nucleotides supplementation ameliorated ethanol-induced developmental defects to some extent and the effects were associated with the dosage of nucleotides. The effect was weak at the dose of 0.16 mg/L, with only scores of flextion, tail neural tube, midbrain, forebrain, and CRL improving compared with ethanol control (*P* < 0.05). 0.80 mg/L group showed significant improvement which was demonstrated as increased levels of all parameters listed in Table 1 except heart and olfactory system. Peak effect was achieved at the dose of 4.00 mg/L with all parameters significantly improved compared with ethanol control (*P* < 0.05). What is more, all the improved parameters except forebrain showed no significant difference in statistics when compared with normal control (*P* > 0.05), which might be considered as strong protective effects against ethanol developmental toxicity. However, when the nucleotides supplementation doses were above 4.00 mg/L, the protective effects became weaker. At the dose of 20.00 mg/L, only scores of flextion, tail neural tube, hindbrain, midbrain, forebrain, auditory system, visual system, and HL increased compared with ethanol control (*P* < 0.05). When the dosage increased to 100.00 mg/L, the scores decreased further and only scores of tail neural tube and forebrain were statistically higher than ethanol control (*P* < 0.05).

### 3.2. Effects of Exogenous Nucleotides on VYS Development of Mouse Embryos Exposed to Ethanol *In Vitro*

**Figure 2:**
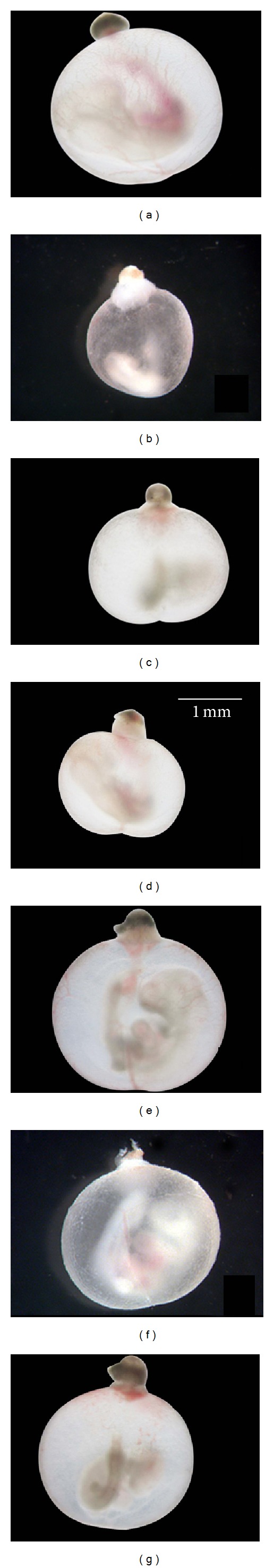
Effects of exogenous nucleotides on VYS development of mouse embryos exposed to ethanol *in vitro*. (a) Normal control; (b) ethanol control; (c) 0.16 mg/L; (d) 0.80 mg/L; (e) 4.00 mg/L; (f) 20.00 mg/L; (g) 100.00 mg/L.

Ethanol-induced toxicity to the yolk sac was detected as shown by reduced diameter and suppressed development of vitelline vessels. A reduced blood/vascular system in ethanol control was also evident by a thinner and less branched network than that of normal control. Nucleotides supplementation showed protective effects to VYS, reflected by increased VYS diameter (*P* < 0.05) (Table 2 and Figure 2) as well as more and thicker vessels in yolk sac compared with ethanol control. 

### 3.3. Effects of Exogenous Nucleotides on Proliferation of MB Cells That Are Exposed to Ethanol

Ethanol-induced proliferation inhibition of MB cells was detected in MTT test. The average OD value of ethanol control group was significantly decreased compared with normal control (*P* < 0.05) (Table 3). Nucleotides supplementation showed protective effects. Similar to WEC results, peak effect was achieved at the dose of 4.00 mg/L, reaching 74.79% of normal control level. The dose of 0.80 mg/L also showed significant protective effect (72.69% of normal control level). The effect was not obvious at the dose of 0.16 mg/L, 20.00 mg/L, and 100.00 mg/L. However 4.00 mg/L and 0.80 mg/L nucleotides supplementation groups did not reach the level of normal control, indicating that under the dosages tested in this investigation, nucleotides supplementation could not completely reverse the proliferation inhibition of ethanol to MB cells. 

### 3.4. Effects of Exogenous Nucleotides on Differentiation of MB Cells That Are Exposed to Ethanol

**Figure 3:**
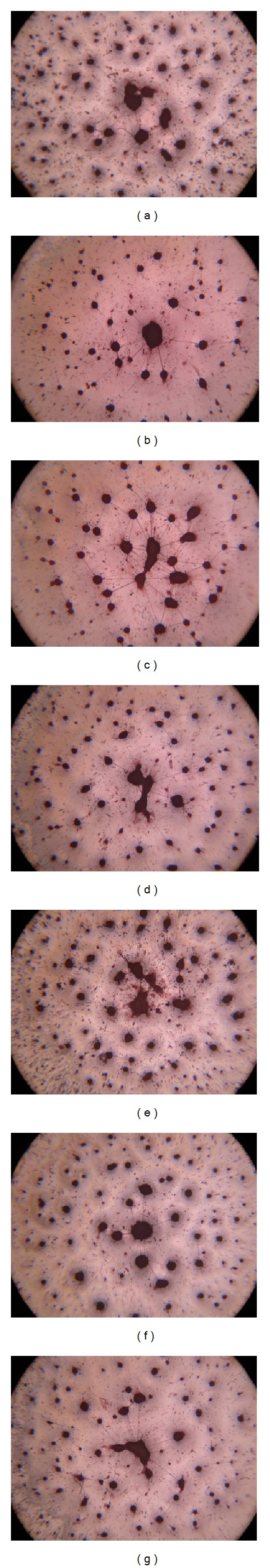
Effects of exogenous nucleotides on differentiation of MB cells that are exposed to ethanol. (a) Normal control; (b) ethanol control; (c) 0.16 mg/L; (d) 0.80 mg/L; (e) 4.00 mg/L; (f) 20.00 mg/L; (g) 100.00 mg/L.

As shown in Table 4 and Figure 3, number of foci was significantly decreased in ethanol control group compared with normal control group. Nucleotides supplementation showed improved results in all the intervention groups, with peak effect achieved at the dose of 4.00 mg/L, whose number of foci reached the level of normal control (*P* > 0.05). 

## 4. Discussion

In this study, we used whole embryo culture (WEC) model and MB cells micromass culture model to explore the effects of exogenous nucleotides supplementation on ethanol-induced embryonic toxicity during the critical organogenesis period and found that certain dose of nucleotides could antagonize the developmental toxicity of ethanol *in vitro*. WEC and Micromass culture models are two useful tools for screening teratogenic and embryo toxic compounds, which are in compliance with 3R principle. The standard protocols are available on the web site of the European Centre for the Validation of Alternative Methods (http://ecvam.jrc.it). Although such *in vitro* experiments have the main disadvantages like the rather short period of embryonic development that can be supported in culture, the present restriction of the techniques to very few species, and the difficulty of mimicking the complicated metabolic situation *in vivo*, they have also shown main advantages such as better animal welfare, allowing precise control of experimental conditions, saving tested chemicals, and being economic to have duplicate tests. Sometimes they can provide information unobtainable from *in vivo* studies. Therefore these methods are now widely used and have proved useful in many studies of normal and abnormal development. 

Results in our present study confirmed ethanol-induced fetal abnormalities and showed that CNS was the main target of ethanol's developmental toxicity, indicated by failure of neural folds to fuse in the midline in one or more regions of the forebrain, midbrain, and hindbrain as well as microcephaly. This was in accordance with other studies and characteristics of FAS [9–11] and was partly explained by the inhibition of MB cells' proliferation and differentiation of ethanol found in micromass test. Developmental abnormalities of other organs such as heart, visual system, and auditory systems shown in our results were also coincided with previous researches [5, 12]. In WEC experiment, the 4 mg/mL ethanol concentration used was determined by our previous experiments [5, 12]. This concentration could induce obvious malformations in the mouse embryo; however was not fatal. According to our previous *in vivo* study in pregnant mice [13], about 4 mg/mL peak level of maternal blood ethanol concentration resulted from a binge drink of 5.0 g/kg ethanol, which was a relatively high ethanol dose in terms of consumption. Since ethanol can cross placenta directly, the ethanol level in amniotic fluid may parallel the blood ethanol level. Therefore we considered that 4 mg/mL of ethanol concentration in this study was equivalent to a high ethanol dose in terms of consumption during pregnancy.

The general belief that cellular nucleotide needs can be met by* de novo* synthesis from nonessential precursors explains why only a limited number of studies have addressed the evaluation of the biological roles of dietary nucleotides. However, our results showed that nucleotides supplementation, especially at the dose of 4.00 mg/L, might be of significant benefit for the development of fetus exposed to ethanol in uterus, especially for CNS. A possible explanation might be that ethanol ingestion induces an increase in purine degradation which decreased nucleotides available for tissues while fetal growth and development calls for increased demand for nucleotides. This might result in nucleotides deficiency in pregnant women and further deficiency in fetus and finally induce fetal malformations. Theoretically, exogenous nucleotides might become a vital source when the metabolic demand exceeds the capacity for endogenous synthesis. A number of recent studies have also shown that exogenous nucleotides status is important for development of body during rapid growth. Animal studies have shown that dietary nucleotides enhance a number of immune responses and the growth, differentiation, and repair of the gut [14–16]. Several clinical studies have reported beneficial effects of nucleotide supplementation on gut microflora, diarrhea, and immune function [17], and the randomized clinical test (RCT) of Cosgrove et al. [18] has reported better catch-up growth in term infants with severe intrauterine growth retardation.

 Another important finding in our experiment is that the protective effect of nucleotide supplementation is associated with the dose of nucleotides. The maximum effect was achieved at 4.0 mg/L, both in WEC and MB micromass culture experiments, and the protection weakened at lower or higher dosage of nucleotides. This is probably due to the mechanism of nucleotide absorption. Proteases and nucleases degrade dietary nucleoproteins and nucleic acids into nucleotides. Intestinal alkaline phosphatases and nucleotidases cleave the phosphate groups from nucleotides to form nucleosides, which are absorbed in the small gut [17]. In mammalian cells, transmembrane flux of nucleosides is mediated by both equilibrative and Na^+^-dependent nucleoside transporters [19]. The equilibrative nucleoside transporters mediate passive downhill transport of nucleosides and function bidirectionally in accordance with the concentration gradient of the substrate. Equilibrative nucleoside transporters exhibit a broad substrate selectivity for both purine and pyrimidine nucleosides and appear to be ubiquitous in mammalian cells. Na^+^-dependent nucleoside transporters mediate active uphill transport of nucleosides into cells by coupling to the inwardly directed Na^+^-gradient across the plasma membrane. Na^+^-dependent nucleoside transporters exhibit distinct transport selectivity for purine and pyrimidine nucleosides. The unique features of Na^+^-dependent nucleoside transporters such as their ability to mediate uphill nucleoside transport, their distinct transport selectivity for purine and pyrimidine nucleosides, and their presence in many critical organs suggest that they may play special physiological and pharmacological roles in mammalian cells [18]. As the kinetic properties of these transporters and their interactions with nucleoside are still not clear, a possible assumption is that transporters were more active at 4.0 mg/L. Higher or lower dose would lower the activity of the transporters and thus decrease the absorption extent of exogenous nucleotides. However, more researches are needed to confirm the assumption, and future study in nucleotides metabolism is needed, including determination of metabolic fate of dietary nucleotides in humans, particularly in pregnant women, bioavailability of nucleotides in mothers to fetus, and the relative contribution of individual nucleotides to observed biologic effects.

Prenatal alcohol exposure has become one of the leading causes of mental retardation in the Western world [20, 21]. On the severe end of the disorder spectrum is fetal alcohol syndrome [1], affecting from 1 to 7 per 1000 live-born infants [22]. The impairments in this syndrome are irreversible; therefore most people with fetal alcohol syndrome are not able to live independently [1], which causes a heavy load for their families and society. As a result, how to intervene ethanol-induced damages has become an emergent public health problem. In the *in vitro* WEC and MB micromass assay, we indicated for the first time that nucleotides supplementation might be of great benefit for fetus exposed to ethanol in uterus. Although results from animal tests may not reflect the exact situation in humans, our research might provide some hints for FAS intervention. Further studies such as randomized clinical tests are required to evaluate the effects of exogenous nucleotides on fetal development.

## 5. Conclusions

The present research indicated that nucleotides supplementation might be of some benefit in the prevention of ethanol-induced birth defects; however appropriate dosage requires attention.

## Figures and Tables

**Table 1:** Effects of exogenous nucleotides on growth and morphology development of mouse embryos exposed to ethanol *in vitro*.

Parameters	Normal control	Ethanol control	Concentration of nucleotides (mg/L)				
			0.16	0.80	4.00	20.00	100.00
CRL	3.63 ± 0.67	3.09 ± 0.75*	3.65 ± 0.51^£^	4.35 ± 0.60^£^	4.08 ± 0.59^£^	3.64 ± 0.45^£^	3.57 ± 0.54^£^
HL	1.98 ± 0.48	1.56 ± 0.41*	1.79 ± 0.28	2.18 ± 0.34^£^	1.99 ± 0.43^£^	1.92 ± 0.31^£^	1.73 ± 0.40
Flextion	4.94 ± 0.25	3.62 ± 0.87*	4.59 ± 0.51^£^	4.83 ± 0.39^£^	4.87 ± 0.52^£^	4.73 ± 0.47^£^	4.58 ± 0.79
Heart	3.75 ± 0.45	2.46 ± 0.52*	2.53 ± 0.51	3.13 ± 0.68	3.33 ± 0.72^£^	2.86 ± 0.78	2.83 ± 0.39
Tail neural tube	5.00 ± 0.00	1.85 ± 0.38*	4.24 ± 0.97^£^	5.00 ± 0.00^£^	5.00 ± 0.00^£^	4.46 ± 0.93^£^	4.33 ± 0.98^£^
Hind brain	4.88 ± 0.34	2.50 ± 0.91*	3.24 ± 0.90	4.58 ± 0.51^£^	4.33 ± 0.82^£^	4.00 ± 0.63^∗£^	3.54 ± 0.96
Midbrain	4.81 ± 0.40	2.04 ± 0.95*	3.59 ± 0.94^∗£^	4.33 ± 0.89^£^	4.27 ± 0.88^£^	4.00 ± 0.77^£^	2.92 ± 0.90
Fore brain	6.00 ± 0.00	1.92 ± 0.76*	3.18 ± 0.98^∗£^	4.50 ± 0.90^∗£^	4.40 ± 0.91^∗£^	4.27 ± 0.90^∗£^	3.17 ± 0.96^∗£^
Auditory system	4.84 ± 0.35	2.35 ± 0.66*	3.35 ± 0.99	4.17 ± 0.72^£^	4.21 ± 0.69^£^	3.91 ± 0.70^∗£^	2.67 ± 0.89
Visual system	4.88 ± 0.34	3.08 ± 0.95*	4.00 ± 0.87	4.83 ± 0.39^£^	4.70 ± 0.46^£^	4.45 ± 0.93^£^	4.17 ± 0.83
Olfactory system	1.88 ± 0.34	0.85 ± 0.38*	0.94 ± 0.24	1.42 ± 0.51	1.79 ± 0.72^£^	1.09 ± 0.83	0.88 ± 0.68
Branchial arch	4.00 ± 0.00	2.92 ± 0.86*	3.41 ± 0.62	3.92 ± 0.29^£^	3.40 ± 0.63^£^	3.36 ± 0.50	3.33 ± 0.65
Forelimb bud	2.72 ± 0.36	2.08 ± 0.40*	2.50 ± 0.47	2.92 ± 0.19^£^	2.73 ± 0.42^£^	2.27 ± 0.52	2.17 ± 0.49
Hindlimb bud	1.75 ± 0.45	0.73 ± 0.63*	1.29 ± 0.85	1.58 ± 0.47^£^	1.67 ± 0.49^£^	1.46 ± 0.69	1.00 ± 0.85

*P < 0.05 versus normal control, ^£^ *P* < 0.05 versus ethanol control.

**Table 2:** Effects of exogenous nucleotides on VYS development of mouse embryos exposed to ethanol *in vitro*.

Parameters	Normal control	Ethanol control	Concentration of nucleotides (mg/L)				
			0.16	0.80	4.00	20.00	100.00
VYS circulation scores	4.09 ± 0.82	1.96 ± 0.92*	2.73 ± 0.69	3.25 ± 0.58^£^	3.70 ± 0.53^£^	3.18 ± 0.98	2.54 ± 0.40
VYS diameter (mm)	4.77 ± 0.55	4.15 ± 0.77*	4.66 ± 0.77^£^	4.67 ± 0.42^£^	4.83 ± 0.62^£^	4.79 ± 0.83^£^	4.78 ± 0.60^£^

*P < 0.05 versus normal control, ^£^ *P* < 0.05 versus ethanol control.

**Table 3:** Effects of exogenous nucleotides on proliferation of MB cells that are exposed to ethanol.

Groups	*N *	OD	Percent of normal control (%)
Normal control	12	0.238 ± 0.034	100.00 ± 0.00
Ethanol control	12	0.149 ± 0.014*	62.61 ± 5.17*
0.16 mg/L NT	12	0.157 ± 0.011*	65.97 ± 4.62*
0.80 mg/L NT	12	0.173 ± 0.012^∗£^	72.69 ± 5.04^∗£^
4.00 mg/L NT	12	0.178 ± 0.009^∗£^	74.79 ± 3.98^∗£^
20.00 mg/L NT	12	0.162 ± 0.010*	68.07 ± 4.20*
100.00 mg/L NT	12	0.157 ± 0.014*	65.97 ± 5.88*

*P < 0.05 versus normal control, ^£^ *P* < 0.05 versus ethanol control.

**Table 4:** Effects of exogenous nucleotides on differentiation of MB cells that are exposed to ethanol.

Groups	*N *	Num. of foci	Percent of normal control (%)
Normal control	8	92.75 ± 3.30	100.00 ± 0.00
Ethanol control	8	56.38 ± 7.31*	60.79 ± 7.88*
0.16 mg/L	8	70.60 ± 6.23^∗£^	76.12 ± 6.72^∗£^
0.80 mg/L	8	76.67 ± 9.20^∗£^	82.66 ± 9.92^∗£^
4.00 mg/L	8	86.57 ± 7.85^£^	93.33 ± 8.46^£^
20.00 mg/L	8	77.25 ± 6.95^∗£^	83.29 ± 7.49^∗£^
100.00 mg/L	8	67.43 ± 7.66^∗£^	72.70 ± 8.26^∗£^

^∗^ *P* < 0.05 versus normal control, ^£^ *P* < 0.05 versus ethanol control.
